# Three Types of Red Beetroot and Sour Cherry Based Marmalades with Enhanced Functional Properties

**DOI:** 10.3390/molecules25215090

**Published:** 2020-11-02

**Authors:** Oana Viorela Nistor, Liliana Șeremet (Ceclu), Gabriel Dănuț Mocanu, Vasilica Barbu, Doina Georgeta Andronoiu, Nicoleta Stănciuc

**Affiliations:** 1 Faculty of Food Science and Engineering, Dunărea de Jos University of Galati, 800201 Galati, Romania; Oana.Nistor@ugal.ro (O.V.N.); Danut.Mocanu@ugal.ro (G.D.M.); Vasilica.Barbu@ugal.ro (V.B.); Georgeta.Andronoiu@ugal.ro (D.G.A.); 2 Faculty of Economics, Engineering and Applied Sciences Cahul State University “B.P. Hasdeu”, 3901 Cahul, Moldova; ceclu.liliana@gmail.com

**Keywords:** red beetroot, sour cherry, marmalade, bioactive compounds, antioxidant activity

## Abstract

The importance of bioactive compounds such as betalains and anthocyanins was highlighted in the present study by the valorization of red beetroot and sour cherry as an attempt to develop healthy products. The aim of the study was to obtain and characterize three types of marmalade based on red beetroot, sour cherry and both in 1:1 combination, obtained by heating at 95 °C for 30 min. Changes in total phenolic content, total flavonoids, betalains, anthocyanins and antioxidant activity were evaluated before and after the thermal treatment. Several other analyses such as color, rheological and textural analyses and confocal laser microscopy were performed to provide further information about the quality of the added-value food products. A significant decrease of 34% in betalains content was registered in the red beetroot marmalade due to the chemical changes in bioactives induced by the temperature. A satisfactory ABTS radical scavenging activity of 8.12 ± 0.38 mMol Trolox/g dry weight (dw) was obtained for the red beetroot and sour cherry based marmalade. The gelled structure was validated by the rheological and textural characteristics. The results highlighted the potential use of red beetroot and sour cherry as food raw materials, due to their particular profile of bioactive compounds.

## 1. Introduction

Fresh fruit and vegetable consumption is increasingly recognized for significant nutritional and functional added-values in the human diet. In the last decades, increased attention has been given to red beetroot due to its nutritional and biological properties. However, due to its specific smell and taste, not all consumers find it pleasant and tasty. Therefore, red beetroot is often combined in the diet with various fruits and vegetables such as carrots, tomatoes, apples, sweet cherries or sour cherries. Red beetroots are consumed in raw form, sometimes shredded as a salad; consumed after thermal processing in which they are grilled, boiled, steamed, roasted, sautéed, canned or even transformed into chips [1]; or introduced as powders into different fortified products.

Beetroot (*Beta vulgaris* L.) originated in Asia and Europe, belonging to the family of Chenopodiaceae [2]. The beetroot is known for its high concentration of bioactive compounds such as betalains, polyphenols, carotenoids and flavonoids, thus providing significant nutritional and health benefits [3]. Probably the most studied bioactive compounds in beetroot, the betalains are classified as betacyanins (responsible for red pigmentation) and betaxanthins (related to yellow color). These are water-soluble nitrogen-containing pigments, found in high concentrations in red beetroot, with reported antimicrobial and antiviral effects [4], potential to inhibit cell proliferation in human tumor cells [5], inhibitory activity towards cervical ovarian and bladder cancer cells in vitro [6], anti-inflammatory effects and antiradical and antioxidant activity [7]. Additionally, red beetroot contains high amounts of vitamins, such as vitamins B, C, A and K, and minerals [8,9].

Bioactive compounds, such as betalains and polyphenols, are widely found in nature, namely in fruits and vegetables. Several epidemiological studies reported that these compounds have the ability to exert biological effects and play a crucial role in preventing diseases linked to oxidative stress. These natural components with antioxidant and antimicrobial properties present great potential in food preservation, gaining relevance in the field of food science and food engineering [10,11]. Red beet and sour cherry are among the most popular natural sources of bioactives owing to not only their nutrient-dense properties, containing betalains and polyphenols, especially anthocyanins, but also their intense aromatic flavor.

The natural pigments from red beetroot are already commercially available as red food colorant recognized by the European Union and Food and Drug Administration (FDA) [12], with multiple uses in nondairy drinks [13], noodles [3], gummy candies, beverages and processed meat products [14].

Sour cherry (*Prunus cerasus* L.), known as tart cherry, is a fruit of the Rosaceae family and is native to northeastern Anatolia [15]. The fruits have a characteristic sour taste and dark red color and are rich in anthocyanins, phenolic acids and flavonoids, such as cyanidin-3-rutinoside, peonidin-3-glucoside, isorhamnetin, quercetin, ferulic acid, chlorogenic acid and *p*-coumaric acid [16]. Due to its rich composition in bioactives, sour cherry is recognized for functional properties in preventing various diseases (preventing neurological diseases, diabetes, obesity and cardiovascular and inflammatory diseases by strong antioxidant, antidiabetic, antiobesity, antimutagenic and anticarcinogenic properties) [17].

Therefore, the aim of this study was to obtain and to characterize a new functional product, namely marmalade, based on red beetroot, sour cherry fruit and agar-agar, with no added sugar. Three different technological variants were analyzed in terms of betalains content, polyphenols, flavonoids, antioxidant activity, color and rheological and textural characteristics. The structure and morphological particularities of the samples were analyzed by confocal electron microscopy. The results highlight the potential use of red beetroot and sour cherry as food raw materials, providing multifunctional properties, due to their particular profile of bioactive compounds.

## 2. Results

### 2.1. The Analysis of Phytochemicals in Fresh, Unprocessed Samples

The phytochemical profiles, in terms of betalains, total monomeric anthocyanins, total polyphenols, flavonoids and antioxidant activity were analyzed by spectrophotometric methods in fresh samples, before thermal processing. The total betalains content in fresh red beetroot samples was 8.35 ± 0.31 mg/g dry weight (dw), which is comparable with the results reported by Castellanos-Santiago and Yahia [18] for red beetroot and Mexican prickly pear, whereas the total polyphenol content (TPC) and total flavonoid content (TFC) were 18.33 ± 0.72 mg gallic acid equivalents (GAE)/g dw and 13.19 ± 0.28 mg quercetin equivalents (QE)/g dw, respectively. Desseva et al. [19] reported values for TPC and TFC of 30.81 ± 2.96 mg GAE/g dw and 6.72 ± 0.16 μg quercetin equivalents/g dw, respectively, in red beetroot juice.

As expected, the fresh sour cherry sample showed a significant content of monomeric anthocyanins (0.64 ± 0.01 mg cyanidin glucoside equivalents—CGE/g dw), whereas TPC (30.17 ± 0.12 mg GAE/g dw) and TFC (21.4 ± 0.04 mg QE/g dw) showed values that were significantly higher (*p* > 0.05) when compared with red beetroot samples. However, no significant differences (*p* < 0.05) were found in antioxidant activities of the fresh samples, thus supporting the hypothesis that anthocyanins and betalains are the main compounds responsible for antioxidant activity of fresh sour cherry and red beetroot samples. When combining red beetroot and sour cherry in a ratio of 1:1, the phytochemical profile revealed an anthocyanin content of 0.61 ± 0.03 mg CGE/g dw. Table 1 shows the physical and phytochemical results of the tested fresh and marmalade samples.

### 2.2. Phytochemical Profile of the Heat Treated Samples

After processing, the marmalade variants were coded, with B representing the red beetroot marmalade, S representing the sour cherry marmalade and BS representing the combination of red beetroot and sour cherry marmalade. The heated samples were treated at 95 °C for 30 min in order to assure the main characteristics and the specific conservation of marmalade. As for the fresh samples, the phytochemicals profiles were analyzed after the thermal processing. Thus, when compared to the fresh samples, the red beetroot marmalade (B) registered a significant decrease of 34% in total betalains content, from 4.34 ± 0.18 mg/g dw to 3.10 ± 0.08 mg/g dw betacyanin and from 4.01 ± 0.03 to 2.43 ± 0.07 mg/g dw betaxanthin. The lower betalains concentration for the processed samples could be due to the isomerization, decarboxylation and/or cleavage of betacyanins during processing [20]. Betacyanins seems to be more heat-stable, with a decrease oapproximately 29%, whereas betaxanthins decreased by more than 39%. Ravichandran et al. [21] showed similar levels of betacyanin and betaxanthin decrease (6%–33%) after the heating of red beetroot at 80 °C for 60, 120 and 180 s. Significant decreases of approximately 60% and 63% were observed in TFC and TPC, respectively.

The monomeric anthocyanins of the sour cherry sample (S) decreased by 28% in the processed samples compared to the fresh ones, whereas TPC and TFC showed decreases of 25% and 49%, respectively, suggesting a stronger thermoprotective effect of the sour cherry matrix.

From Table 1, it can be observed that the food matrix obtained from the combination of red beetroot and sour cherry showed a more protective effect on bioactives, with decreases in TPC and TFC of 35% and 30%, respectively, whereas the anthocyanin concentration was by approximately 50%. Betalains seemed to be more stable, with betacyanin and betaxanthin concentrations of 0.83 ± 0.024 and 1.04 ± 0.03 mg/g dw, respectively, in the processed food.

The most important reduction in antioxidant activity was found for red beetroot and was correlated with the decrease in TPC and TFC. Good preservation of ABTS radical scavenging activity was observed for the processed sour cherry marmalade, with a decrease of approximately 36% during thermal processing. An explanation could be related to the possible presence of polyhydroxylated and polymethoxylated glycosides [22], which are produced by the anthocyanin polymerization processes as an effect of the thermal treatment [23]. The newly made marmalade product, obtained by combining sour cherry and red beetroot (BS), showed a satisfactory ABTS radical scavenging activity of 8.12 ± 0.38 mMol Trolox/g dw. The antioxidant activity (determined by the DPPH method) varied between 0.87 ± 0.07 and 2.75 ± 0.02 μMol Trolox/g dw for the marmalade samples, with the lowest values being measured in the B sample. Similar to the results obtained by ABTS assay, the antioxidant activity was influenced by the thermal treatment (Table 1).

### 2.3. Water Activity (a_w_) of the Samples

It is well known that long-term storage stability is achieved through a combination of the equilibrium between thermal processing and control of water activity. After the composition, boiling is the most important process in marmalade making. Thus, the samples were processed by using a Multicooker. Due to the hermetic seal of the equipment, an increase in vapor pressure can be achieved, ensuring that the product is not affected by the caramelization process.

The values obtained for the water activity of the marmalade samples were in the range of 0.83 to 0.86 (Table 1). These values are important for canned foods, considering that the minimum a_w_ level for the growth of *Clostridium botulinum* is approximately 0.93.

### 2.4. Color Measurement

Colors are important quality indicators that determine the consumer acceptance of foods. Table 2 shows the color parameters of the marmalade samples.

The values of the L* parameter ranged between 21.5 ± 0.28 and 29.66 ± 0.02. The processed products were darker compared to the fresh samples (lower L* values), probably due to non-enzymatic browning determined by sugar caramelization or Maillard reactions (data not shown). Similar results were obtained by Igual et al. [24] in the case of grapefruit jams. The processing procedure decreased a* value. The B marmalade was less reddish when compared with S and BS samples due to the presence of anthocyanins. Anthocyanins are reported to have a significant role in the color quality of many fruits. The yellow-blue coordinates (b*) were higher for S and BS, ranging from 7.40 ± 0.09 (S) to 4.29 ± 0.14 (B). Throughout the processing period, the proportion of yellow color increased due to the temperature and cooking time when compared to the control samples. Rababah et al. [25] observed an increase in the b* parameter when examining cherry jams. The color of the marmalades revealed that the total color difference (Δ*E*) was higher in the case of the S sample (7.01 ± 0.23). The major causes of color change were probably because of the browning reactions. Chroma (C*), the measure of color saturation, decreased in the case of processed samples, which indicates that the color of these samples is not so intense. The Chroma values of all the processed samples were between 15.62 ± 0.12 (B) and 23.67 ± 0.09 (BS). The hue angle (h*), which describes the color wheel/cycle, of all of the samples ranged from 15.94 ± 0.48 (B) to 21.89 ± 0.11 (S).

### 2.5. Rheological Measurements Results

Viscosity curves for the tested marmalades were generated by evaluating the strain from 0.01% to 100% using geometry with grooved surfaces to avoid apparent wall slip. When the structure of the samples is not affected during the test, *G’* and *G”* relate to the “solid-like” and “liquid-like” properties of the samples, respectively [26].

The low amplitude oscillatory measurements revealed a good resistance of sour cherry marmalade to the applied strain, with *G’*–*G”* intersection point at ~26% strain. This compensated for the poor rheological properties of B-flow point at 4% strain and narrow LVR, for the marmalade obtained by mixing the two ingredients (BS). However, B presented the highest consistency, with *G’* values up to 7 kPa. *G’* registered greater values than *G”* at any given point in the frequency sweep tests; this is typical behavior for gels with a predominantly elastic character. For all the marmalade samples, the slip effects occurred for both the smooth and grooved geometries. These may be due to the presence of the fibers, which are present in all samples, similar to the results obtained by Barbieri et al. [27] for the pulp and jam of gabiroba.

**Figure 1:**
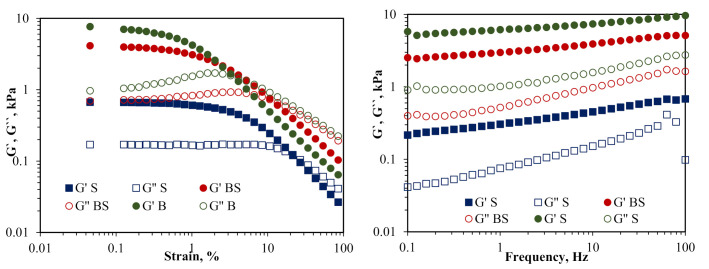
Elastic modulus (G’) and viscous modulus (G”) variation with stain and frequency (B, red beetroot marmalade; S, sour cherry marmalade; BS, red beetroot and sour cherry marmalade).

The dynamic viscoelastic properties of marmalades were evaluated by elastic (*G’*) and viscous (*G”*) moduli as a function of frequency (Figure 1). Both moduli had a slight frequency dependence, with *G’* exceeding *G”* at all the frequencies analyzed (0.1–100 Hz).

The results are in accordance with those of Basu et al. [28] for mango jam and Gabriele et al. [29] for jam and yoghurt. Considering these results, the *G’* and *G”* values of each marmalade sample were averaged and represented as a function of the angular frequency. It seems that the *G’* of all samples increased with the frequency, which indicates a relaxation process of the marmalade matrix. However, the values of *G’* and *G”* seem to be not parallel, so the samples are considered strong gels, in contradiction with the findings of Figueroa et al. [30] for fruit jellies enriched with dietary fiber. All three samples presented a low frequency dependence with no *G’–G”* intersection point over the entire tested frequency range. 

### 2.6. Texture Analysis Results

Table 3 shows that all the values of all textural parameters for the BS sample are between the values of the other two samples.

From Table 3 it can be seen that the highest firmness (2.99 N) was registered for beetroot marmalade, while the lowest firmness (1.53 N) was registered for sour cherry marmalade. The combined marmalade had an intermediate value of 2.09 N. This behavior is due to the vegetal material microstructure: the fibrous tissue fragments from beetroot determine a high resistance to penetration. Similar firmness values were reported by Banas et al. [31] for low-sugar gooseberry jams enriched with plant ingredients and by Garrido et al. [32] for apple jellies.

Adhesiveness is a measure of the work required to withdraw the probe from the sample. In our study, these parameters registered values between 1.17 mJ for the S sample and 5.26 mJ for the B sample. The most cohesive sample was the S sample. The mixing of red beetroot with sour cherries resulted in weakening the bonds between the structural elements of the sample. A similar effect was noticed for springiness.

### 2.7. Structural Particularities of the Marmalades

**Figure 2:**
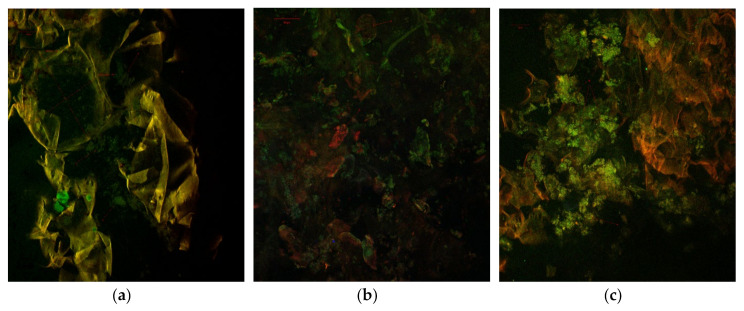
The confocal laser scanning microscopy images for the marmalade samples: (**a**) red beetroot, (**b**) red beetroot and sour cherry and (**c**) sour cherry.

The confocal scanning microscopy revealed the presence of selected biologically active compounds, such as betalains (Bt) in red beetroot marmalade and anthocyanins (An) in sour cherry marmalade (Figure 2).

Similar results are reported by Miguel [33] and Cabrera-Bañegil et al. [34]. The compounds were aggregated in clusters of variable sizes, which could be due to the agar-agar addition. The structural and biochemical characteristics of both vegetal tissues could explain the differences in cellular wall behavior in response to the same thermal treatment. The cells from the sour cherry marmalade were completely lysed, more than those of red beetroot. The marmalade made from or with red beetroot contained more or less undamaged tissue fragments belonging to the root parenchyma with cells of 233.03–269.49 µm.

## 3. Materials and Methods

### 3.1. Chemicals

Agar-agar (Biovegan, Bonefeld, Germany), ethanol, Folin-Ciocalteu’s reagent, sodium carbonate, ABTS (2,2-azino-bis(3-ethylbenzothiazoline-6-sulfonic acid) diammonium salt), quercetin, methanol, aluminum chloride, potassium chloride, sodium acetate and HCl were purchased from Sigma Aldrich (MilliporeSigma, Steinheim, Germany).

### 3.2. Plant Material

Fresh red beetroot (*Beta vulgaris* L.) and frozen sour cherries (*Prunus cerasus* L.) were purchased from a local market (Carrefour) from Galati, Romania between February and March 2020. Fresh red beetroot was washed, peeled, cut into cubes and stored at −20 °C.

### 3.3. Marmalade Making

Three variants of marmalade were obtained based on frozen red beetroot, sour cherry and a combination of both of them in a ratio of 1:1. Frozen samples were mixed with 30% distilled water and 1% agar-agar (Biovegan, Germany). The mixture was blended for 10 min at 1000 rpm with a Philips HR2100/40 blender, EC (European Community). The mixture was heated in a Multicooker (Philips HD3037/70, 980 W, 5 L, Eindhoven, the Netherlands) at a special program for jam manufacturing (95 °C for 30 min). The marmalades were processed until the dry weight was doubled. The marmalades were packed in jars and equilibrated at room temperature (22 °C) and then stored at 4 °C for 1 week before analysis. The variants were coded as follows: B, the red beetroot marmalade; S, the sour cherry marmalade; BS, the combination of red beetroot and sour cherry marmalade.

### 3.4. Analysis of Betalains

The betalain content was analyzed as described by Ravichandran et al. [20]. One gram of sample was dissolved in 10 mL of 20% ethanol and mixed for 10 s, and then the homogenate was centrifuged at 6000× g for 10 min. The centrifugation was repeated 2 times to ensure maximum extraction of betalains. The supernatant was used for the determination of betalains. The content of betaxanthins and betacyanins in the extracts was determined spectrophotometrically at 538 and 480 nm with a UV–Vis spectrometer [35]. The betalain content (BC) was calculated according to Equation (1):

(1) $$BC=A\cdot D_{f}\cdot M_{W}\cdot \frac{V_{d}}{\varepsilon }\cdot L\cdot W_{d},\;mg/g\;$$

where *A* is the absorbance, *D_f_* is the dilution factor, *V_d_* is the solution volume in milliliters, *W_d_* is the sample weight in grams and *L* is the path length (1 cm) of the cuvette. For quantification of betacyanins the molecular weight (*M_W_*) and molar extinction coefficient (*ε*) of *M_W_* = 550 g/mol and *ε* = 60,000 L/(mol cm) were applied; for betaxanthins, these values were *M_W_* = 308 g/mol and *ε* = 48,000 L/(mol cm).

### 3.5. Total Anthocyanin Content

A volume of 3 mL of extract was diluted in 5 mL of two different buffers: 0.025 M potassium chloride, pH = 1.0, and 0.4 M sodium acetate, pH = 4.5. After 30 min of incubation at room temperature, absorption (A) was measured at λ = 510 and 700 nm. All extracts were analyzed in triplicate. For calculation of total anthocyanins as cyanidin glucoside equivalents—CGE, the molar absorptivity coefficient (ε) values 26,900 M^−1^cm^−1^ and the molecular weight of 449 Da were used [36].

The results were calculated similarly to [37] as follows:

Asp = (A510 − A700) pH1.0 − (A510 − A700) pH 4.5

The content of total anthocyanins (TA) were calculated as follows:

TA = (Asp × M × *D_f_* × 1000) / (ε× λ × m) (2)

where *D_f_* is the dilution factor, λ is the cuvette optical path length (1 cm) and m is the weight of the sample in grams. The total anthocyanin content was expressed as mg CGE/g dw.

### 3.6. ABTS Radical Scavenging Assay

The fresh ABTS (2,2-azino-bis(3-ethylbenzothiazoline-6-sulfonic acid) diammonium salt) solution was diluted with ethanol (96%) until the absorbance of 0.700 ± 0.02 at 734 nm. Then, 10 µL of the extract was added to 1 mL of the ABTS radical solution and shaken for 10 s. One minute after the addition of the sample, the decolorization that was caused by the reduction of the cations by the antioxidants from the sample was measured spectrophotometrically at 734 nm (Biochrom Libra S22 UV/Vis, Cambridge, United Kingdom). The experiments were performed in triplicate. A standard curve using Trolox was used to express the antioxidant activity as mMol Trolox equivalent/g dw. The experiments were performed in triplicate.

### 3.7. DPPH Radical Scavenging Assay

The antioxidant activity of the samples was determined as a measurement of radical scavenging using the DPPH radical. Thus, 100 µL of a marmalade aqueous extract sample was mixed in triplicate with 3.0 mL of a DPPH work solution in absolute methanol. The mixture was incubated for 120 min in the dark at room temperature. The absorbance was measured at 515 nm against absolute methanol. For the control sample, the 100 µL of marmalade aqueous extract was replaced with 100 µL of absolute methanol. The results of the assay were expressed as Trolox equivalents (mM Trolox/g dw). The experiments were performed in triplicate.

### 3.8. Water Activity

The water activity of the samples was measured with a LabSwift water activity instrument (Novasina, Lachen, Switzerland). The experiments were performed in triplicate.

### 3.9. Total Polyphenol Content (TPC)

Five hundred microliters of the extract from each sample was introduced into test tubes and mixed with 2.5 mL of a tenfold dilute Folin–Ciocalteu reagent and 2 mL of 7.5% sodium carbonate. The tubes were covered with aluminum foil and allowed to stand for 30 min at room temperature before the absorbance was read at 765 nm using a UV–Vis spectrophotometer (Biochrom Libra S22 UV/Vis, Cambridge, United Kingdom) [38]. Each assay was performed in triplicate. The results were expressed as milligrams of gallic acid equivalents per gram dry weight (mg GAE/g dw).

### 3.10. Total Flavonoid Content (TFC)

The aluminum chloride colorimetric method was used for the determination of the total flavonoid content of the sample. For total flavonoid determination, quercetin was used to make the standard calibration curve. Stock quercetin solution was prepared by dissolving 5.0 mg quercetin in 1.0 mL methanol, then the standard solutions of quercetin were prepared by serial dilutions using methanol (5–200 μg/mL). An amount of 0.6 mL diluted standard quercetin solutions or extracts was separately mixed with 0.6 mL of 2% aluminum chloride. After mixing, the solution was incubated for 60 min at room temperature. The absorbance of the reaction mixtures was measured against blank at 420 nm wavelength with a UV–Vis spectrophotometer (Biochrom Libra S22 UV/Vis, Cambridge, United Kingdom). The concentration of total flavonoid content in the test samples was calculated from the calibration plot and expressed as mg quercetin equivalent (QE)/g dw [39]. All the determinations were carried out in triplicate.

### 3.11. Color Measurement

The color was measured using a Minolta Chroma Meter CR-410 (Konica Minolta, Osaka, Japan) fitted with a granular accessory, after standardization with a white calibration plate according to the equipment specifications. The determined parameters were L* (lightness/darkness), a* (red/green) and b* (yellow/blue). The total color difference (Δ*E*) between samples was calculated according to Equation (3):

(3) $$∆E=\;\sqrt{{\left(L_{0}^{*}-\;L^{*}\right)}^{2}+{\left(a_{0}^{*}-a^{*}\right)}^{2}+{\left(b_{0}^{*}-b^{*}\right)}^{2}}$$

Subscript 0 refers to the color of the fresh sample. The hue angle (h*), visual color appearance, chroma (C*) and color intensity were calculated according to Equations (4) and (5):

(4) $$C^{*}=\sqrt{a^{*2}+b^{*2}}$$

(5) $$h^{*}=\;tan^{-1}\left(\frac{b^{*}}{a^{*}}\right)$$

Three replicates were carried out for each sample.

### 3.12. Rheological Measurements

The rheological behavior of marmalade samples was determined with a controlled stress rheometer AR2000ex (TA instruments Ltd., New Castle, Delaware, USA) equipped with a Peltier plate for temperature control and a plate geometry of 40 mm in diameter, with a set gap of 2 mm. The system temperature was set to 20 °C. Low-amplitude dynamic shearing tests of strain sweep and frequency were applied, and storage modulus (G’) and loss modulus (G’) were registered. The strain sweep test was performed at an oscillatory frequency of 1 Hz while increasing strain from 0.01% to 100%. It was used to identify the linear viscoelastic region (LVR) for all investigated samples. The dynamic frequency sweeps were further performed in the 0.1–100 Hz domain at a constant strain of 0.2% (determined to be within the linear viscoelastic region). The experiments were performed in triplicate.

### 3.13. Texture Analysis

The textural parameters (firmness, adhesiveness, cohesiveness and springiness) were determined with a Brookfield CT3 texture analyzer (AMETEK Brookfield, Middleboro, Massachusetts, USA). The samples were collected immediately after manufacturing and packed into cylindrical plastic containers, with 30 mm diameter and 50 mm height. The containers were kept at 4 °C for 1 week, and before testing they were equilibrated at room temperature for 3 h. A double penetration test was applied, using a 25.4 mm acrylic cylinder. The testing parameters were set as follows: target distance 10 mm, trigger load 0.067 N, pretest speed 2 mm/s, test speed 1 mm/s, return speed 1 mm/s, load cell 1000 g. The textural parameters were determined with TexturePro CT V1.5 software, provided by Brookfield Engineering Labs, Inc., AMETEK Brookfield, Middleboro, Massachusetts, USA. Five determinations for each sample were made and the presented results are the means of these determinations.

### 3.14. Confocal Laser Scanning Microscopy (CLSM)

The aim of the CLSM analyses was to highlight the size and morphology of the three variants of marmalade with red beetroot and sour cherry. For this study, a Zeiss LSM 710 confocal laser system (Carl Zeiss MicroImagining, Göttingen, Germany) was used to capture the 3D images, which were analyzed by the ZEN 2012 SP1 software (black edition). The technical specifications of the equipment used are as follows: diode laser (405 nm), Ar laser (458, 488, 514 nm), diode-pumped solid-state (DPSS) laser (561 nm) and HeNe laser (633 nm); AxioObserver Z1 inverted microscope, 63× apochromatic objective (numerical aperture 1.4); FS49, FS38 and FS15 filters. To capture the autofluorescence of the samples, the emission was measured at a wavelength between 405 and 633 nm. In order to acquire the images, the microparticles were stained with two dyes, DAPI (1 μg/mL) and Red Congo (40 μM), in a ratio 3:1:1. The acquisition parameters of the images were as follows: line scan mode, mean method, speed 6, 12-bit depth. In order to increase the signal-to-noise ratio, the frame average of eight scans was used.

### 3.15. Statistical Analysis

All experimental measurements were performed at least in triplicate, and the results are presented as mean value ± standard deviation (SD). The one-way analysis of variance (ANOVA) and Tukey’s test with a 95% confidence interval were applied using Minitab 18 software–Free Trial (Ottawa, ON, Canada) to identify significant differences.

## 4. Conclusions

The results obtained in this study emphasize the potential of red beetroot and sour cherry to be used in developing processed foods with no added sugar. This study was focused on evaluating the stability of total phenolics, flavonoids, anthocyanins and betalains of red beetroot and sour cherry in fresh products and marmalades during processing in relation to antioxidant capacity. A significant effect of processing on selected phytochemicals was observed for all samples. When combining red beetroot and sour cherry, a protective effect was evidenced whereby the complex matrix prevented the thermal degradation of phenolic and flavonoids, whereas the anthocyanin concentration was reduced by approximately 50%. Betalains were more heat stable, leading to good preservation of the antioxidant activity. The different thermal behaviors of the bioactives were highlighted by confocal scanning microscopy, showing selected phytochemicals such as betalains and anthocyanins aggregated in clusters of variable sizes, with the cells from the sour cherry marmalade more damaged when compared with those of red beetroot. As expected, red beetroot marmalade showed the highest consistency, with a typical behavior for gels, having a predominantly elastic character.

More studies are needed in order to test the bioavailability of bioactives during digestion and the effect of storage on the preservation of microbial safety in marmalades before the production of these products at an industrial scale. In addition, a sensorial analysis is planned by our research group, in order to test the acceptability of the products.

## Figures and Tables

**Table 1:** Physical and phytochemical results of the tested fresh and marmalade samples.

Code	a_w_	TPC, mg GAE/g dw	TFC, mg QE/g dw	ABTS Radical Scavenging Activity, mMol Trolox/g dw	DPPH, µM Trolox/g dw	Betalains		TAC, mg C3G/g dw
						β-Cyanin, mg/g dw	β-Xanthin, mg/g dw	
B_0_	0.94 ± 0.02 ^a^	18.33 ± 0.72 ^a^	13.19 ± 0.28 ^b^	13.10 ± 2.31 ^a^	1.80 ± 0.04 ^a^	4.34 ± 0.18 ^a^	4.01 ± 0.03 ^a^	-
B	0.86 ± 0.01 ^b^	6.88 ± 0.22 ^b^	5.33 ± 0.08 ^c^	3.88 ± 0.57 ^c^	0.87 ± 0.07 ^c^	3.10 ± 0.08 ^b^	2.43 ± 0.07 ^b^	-
S_0_	0.98 ± 0.01 ^a^	30.17 ± 0.12 ^a^	21.4 ± 0.04 ^b^	17.77 ± 0.16 ^a^	4.30 ± 0.05 ^a^	-	-	0.64 ± 0.01 ^a^
S	0.86 ± 0.02 ^b^	22.78 ± 1.19 ^b^	10.93 ± 0.51 ^c^	11.38 ± 0.42 ^c^	2.75 ± 0.02 ^c^	-	-	0.46 ± 0.08 ^b^
BS_0_	0.96 ± 0.01 ^a^	20.12 ± 0.42 ^a^	13.86 ± 0.16 ^b^	14.22 ± 0.13 ^a^	2.53 ± 0.03 ^a^	1.27 ± 0.03 ^a^	1.38 ± 0.02 ^a^	0.61 ± 0.03 ^a^
BS	0.83 ± 0.01 ^b^	13.01 ± 0.39 ^b^	9.83 ± 0.45 ^c^	8.12 ± 0.38 ^c^	1.44 ± 0.04 ^c^	0.83 ± 0.024 ^b^	1.04 ± 0.03 ^b^	0.31 ± 0.09 ^b^

a_w_, water activity; TPC, total phenolic content; TFC, total flavonoid content; TAC, total anthocyanin content; B_0_, fresh red beetroot; S_0_, fresh sour cherry; BS_0_, fresh red beetroot and sour cherry; B, red beetroot marmalade; S, sour cherry marmalade; BS, red beetroot and sour cherry marmalade. Values are represented as mean ± standard errors. Different superscript letters (a, b, c) mean a significant difference at (*p* < 0.05) among different parameters for the same column.

**Table 2:** Color parameters of marmalades.

Color Parameters	Marmalades		
	B	S	BS
L* (clarity)	21.50 ± 0.28 ^a,^ *	29.66 ± 0.02 ^b^	25.32 ± 0.05 ^b^
a* (red/green color component)	15.02 ± 0.21 ^a^	18.41 ± 0.17 ^b^	22.46 ± 0.08 ^b^
b* (blue/yellow color component)	4.29 ± 0.14 ^a^	7.40 ± 0.09 ^b^	7.50 ± 0.04 ^b^
Δ*E* (total color difference)	7.01 ± 0.23 ^a^	6.46 ± 0.10 ^a^	5.48 ± 0.05 ^a^
C* (chroma)	15.62 ± 0.12 ^a^	19.84 ± 0.19 ^b^	23.67 ± 0.09 ^b^
h* (hue angle)	15.94 ± 0.48 ^a^	21.89 ± 0.11 ^b^	18.46 ± 0.04 ^a^

Values are represented as mean ± standard errors. Different superscript letters (a and b) mean a significant difference at (*p* < 0.05) among different parameters on the same row. * Standard deviations values.

**Table 3:** Values of marmalade textural parameters.

Texture Parameter	Sample		
	B	S	BS
Firmness, N	2.99 ± 0.07 ^a,^*	1.53 ± 0.08 ^b^	2.09 ± 0.07 ^b^
Adhesiveness, mJ	5.26 ± 0.88 ^a^	1.18 ± 0.03 ^b^	4.73 ± 0.17 ^a^
Cohesiveness, -	0.32 ± 0.01 ^b^	0.65 ± 0.05 ^b^	0.36 ± 0.01 ^b^
Springiness, mm	6.67 ± 0.62 ^a^	8.85 ± 0.57 ^a^	7.53 ± 0.52 ^a^

* Standard deviations; B, red beetroot marmalade; S, sour cherry marmalade; BS, the combination of red beetroot and sour cherry marmalade. Values are represented as mean ± standard errors. Different superscript letters (a and b) mean a significant difference at (*p* < 0.05) among different parameters on the same row.
